# Relationship between sleep parameters, insulin resistance and age-adjusted insulin like growth factor-1 score in non diabetic older patients

**DOI:** 10.1371/journal.pone.0174876

**Published:** 2017-04-06

**Authors:** Sarah Damanti, Olivier Bourron, Mohamed Doulazmi, Anne-Laure Mandengue Sosso, Vi-Huong Nguyen-Michel, Jean Mariani, Kiyoka Kinugawa

**Affiliations:** 1 Assistance Publique-Hôpitaux de Paris (APHP), DHU FAST, Functional Exploration Unit of older patients, Groupe hospitalier Pitié-Salpêtrière-Charles Foix, Paris, France; 2 Sorbonne Universités, UPMC Univ Paris 06, UMR 8256, Biological Adaptation and Aging, Paris, France; 3 Università degli Studi di Milano, Milan, Italy; 4 Assistance Publique-Hôpitaux de Paris (APHP), Diabetology Department, Pitié Salpétrière Hospital, Paris, France; 5 Sorbonne Universités, UPMC Univ Paris 06, Paris, France; 6 INSERM, UMR_S 1138, Centre de recherche des Cordeliers, Paris, France; 7 CNRS, UMR 8256, Biological Adaptation and Aging, Paris, France; Centre de Recherche des Cordeliers, FRANCE

## Abstract

**Background:**

Sleep complaints are prevalent in older patients. Sleepiness, short or long sleep duration and obstructive sleep apnea (OSA) are associated with insulin resistance (IR). These parameters have not yet been considered together in the same study exploring the possible association between IR and sleep in older patients. IR is involved in cardiovascular and metabolic diseases, pathologies which are highly prevalent in older patients. Here we assess, in older non-diabetic patients with sleep complaints, the associations between IR and sleep parameters objectively recorded by polysomnography (PSG) rather than self-report. The Growth Hormone/Insulin like growth factor-1 axis could play a role in the development of IR during sleep disorders. The second objective of this study was to analyze the association between sleep parameters and age-adjusted IGF-1 score, which could explain the association between OSA and IR.

**Methods:**

72 non-diabetic older patients, mean age 74.5 ± 7.8 years, were included in this observational study. We evaluated anthropometric measures, subjective and objective sleepiness, polysomnography, Homeostatic Model Assessment for IR (HOMA-IR) and age-adjusted IGF-1 score. A multivariate regression was used to determine factors associated with HOMA-IR.

**Results:**

The 47 OSA patients were over-weight but not obese and had higher IR than the non-OSA patients. In multilinear regression analysis, apnea-hypopnea index was independently associated with IR after adjustment for several confounding factors. Neither IGF-1 level nor IGF-1 score were different in the two groups.

**Conclusions:**

We demonstrate that in non-diabetic older patients with sleep complaints, OSA is independently associated with IR, regardless of anthropometric measurements and sleep parameters (sleep duration/sleepiness/arousals). Targeting OSA to reduce IR could be useful in the elderly, although further exploration is required.

## Introduction

Sleep complaints and sleep disorders, including obstructive sleep apnea (OSA) [1] are progressively prevalent conditions in older people. OSA is characterized by repetitive upper airway obstruction during sleep resulting in apneas and hypopneas with subsequent intermittent hypoxia, sleep fragmentation and diurnal sleepiness. Focusing on OSA is important because it is associated with cardiovascular and metabolic diseases in both middle-aged and older adults [2]. Insulin resistance (IR), characterized by decreased cellular response to insulin, is among the metabolic disorders associated with OSA. IR contributes to the development of diabetes and is a major risk factor for cardiovascular diseases [3]. Cross–sectional studies have shown that OSA impairs glucose tolerance and/or insulin sensitivity, as measured by Homeostasis Model Assessment-Insulin Resistance index (HOMA-IR), even after adjusting for body mass index (BMI) and even in non-diabetic patients [4,5]. IR, glucose intolerance and type 2 diabetes prevalences are reported between 20% to 67% higher in OSA patients than in controls [6]. Moreover, the severity of nocturnal hypoxia in non-obese OSA patients is associated with IR, suggesting that intermittent hypoxia plays a major role in this metabolic dysfunction [7,8]. The strong link between OSA and IR is also reinforced by the effectiveness of the OSA treatment with continuous positive airway pressure (C-PAP) in improving IR in middle-aged people [9]. However, these analyzes, though showing the strong association between OSA and IR, did not take into account other potential confounding factors, such as duration, quality and fragmentation of sleep, snoring and daytime sleepiness as suggested by other studies [10,11]. Indeed, in large population-based studies in middle-aged individuals without diabetes, subjective sleep complaints, self-reported sleep duration and daytime napping have been associated with IR, though without an objective OSA diagnosis by nocturnal recording [12,13]. Also in non-diabetic older subjects, self-reported symptoms of sleep-disordered breathing are associated with IR and increased incidence of type 2 diabetes [11]. Thus it is not clear if the association between OSA and IR is due to apnea-hypopneas index (AHI), hypoxia severity, sleep duration, sleepiness or other sleep parameters.

The purpose of the present analysis was to assess, in non-diabetic older outpatients consulting for sleep complaints, the associations between IR and sleep parameters objectively recorded by polysomnography (PSG) rather than by self-report. Insulin like growth factor 1 (IGF-1) plays a key role in glucose homeostasis by stimulating peripheral glucose uptake and reducing hepatic glucose production. A reduction of IGF-1 level contributes to a decrease in insulin sensitivity [14]. Sleep disorders and OSA may affect the Growth Hormone/Insulin like growth factor 1 (GH/IGF-1) axis with an inverse relationship between IGF-1 plasma concentration and the severity of OSA, which could be the mechanism involved in the association between OSA and IR [15,16]. Considering the influence of age on the GH/IGF-1 axis, a previous study has focused on the relationship between sleep, OSA and GH/IGF-1 in older subjects [17] but showed no significant relationship. To account for the influence of age on IGF-1 axis, an age-adjusted IGF-1 score has previously been proposed [18]; the second objective of this study was to analyze the association between sleep parameters and this age-adjusted IGF-1 score, to determine whether this is the mechanism underlying the association between OSA and IR.

## Methods

### Participants

72 community-dwelling outpatients, aged ≥60 years, referring for sleep disorders in the Functional Explorations and Sleep Investigation Unit for the Elderly of the university geriatric hospital in the Pitié-Salpêtrière-Charles-Foix group (Pierre et Marie Curie University), were included. The study was part of a systematic medical assessment and healthcare program for older adults referred to our sleep clinic center. All participants received a letter informing them of the sleep assessment procedures (scales, cognitive tests, polysomnography, multiple sleep latency tests, blood analysis) and that their results would be anonymized and stored for research. On the basis of this program, the institutional review board of the local Ethics Committee Paris 6 approved the study protocol, considering the full study as an acceptable variant of routine care procedures and determined that individual written consent could be waived. A sleep diary for at least seven days was obtained. Clinical assessment included: sleep habits and medications, anthropometric measures (weight, height, BMI, waist, neck and hip circumferences), Epworth Sleepiness Scale (ESS), Geriatric Depression Scale (GDS), Mini-Mental State Examination (MMSE), overnight home PSG and on the following day in the sleep laboratory, blood tests and multiple sleep latency tests (MSLT) (four consecutive tests starting two hours after awakening, at two-hour intervals). Standard meals were provided without alcohol or caffeine. Exclusion criteria were: diabetes, glycated hemoglobin ≥ 6.5% (47.5 mmol/mol), fasting glycemia >7mM, C-PAP or oxygen treatment, PSG recording time < 180 minutes, central sleep apnea, missing data for fasting insulin, glucose or HOMA-IR.

### Technical measurements

#### Polysomnography

Sleep recording was performed using an ambulatory PSG (Trackit™ 18+8, Lifelines Ltd, UK) measuring the following variables: electroencephalography (EEG) (C4-A1, C3-A2, O1-A2, O2-A1, Fp1-Cz, Fp2-Cz), electrooculogram, chin and leg electromyogram, nasal cannula/pressure transducer linked to a breathing noise transducer, thoracic-abdominal respiratory effort, pulse oximeter, electrocardiogram, and position sensor. Sleep stages were assigned according to published guidelines [19]. Apneas were defined as a complete absence of nasal airflow for at least 10 seconds, accompanied by either persistent (obstructive apnea) or abolished thoracic-abdominal movements (central apnea). Hypopneas were defined as events lasting at least 10 seconds with either a ≥ 50% decrease in the airflow or a ≥ 30% decrease in airflow accompanied by a ≥ 3% decrease in oxyhemoglobin saturation or an EEG arousal. A flow limitation was a decrease of 30% or less in the form of classic flattening of the airflow limb for at least 2 consecutive breaths, lasting ≥ 10 seconds and ending in an EEG arousal. AHI was defined by the sum of apneas, hypopneas and flow limitations per hour of sleep. OSA was diagnosed if AHI was at least 15/hr. Moderate OSA was defined between 15 and 30/hr and severe as AHI ≥30/hr.

#### Biochemical analyzes

Venous blood was sampled after 12 hours overnight fasting to measure: fasting blood glucose, glycated hemoglobin, total-, high density lipoprotein (HDL) -, and low density lipoprotein (LDL) -cholesterol, triglycerides, hepatic function (alanine aminotransferase (ALT), aspartate aminotransferase (AST), gamma-glutamyl transferase (GGT)), thyroid function (thyroid stimulating hormone (TSH), free triiodothyronine (fT_3_), free thyroxine (fT_4_), creatinine, nutritional indices (proteinaemia, albumin, prealbumin, transferrine, phosphate, lymphocytes). Plasma insulin and IGF-1 concentration were determined by chemiluminescence. HOMA-IR was used to estimate IR from a mathematical model (HOMA-IR = [(fasting glycaemia)x(fasting insulinemia)]/22.5), closely correlated to the gold standard invasive technique of euglycemic clamp [20]. Based on the traditional definition of insulin resistance (HOMA-IR ≥2.5), we delineated 2 HOMA-IR categories prior to further analyses: <2.5, and ≥2.5 [20]. IGF-1 score was calculated according to the formula IGF-1 score = {[(log IGF-1+0.00625 x age)—2.555] / 0.104}. IGF-1 was expressed in μg/L [18].

#### Statistical analysis

We compared the OSA group to the non-OSA group. Categorical variables were compared using Pearson’s χ^2^ test or Fisher’s exact test. Quantitative variables were analyzed using Student’s t-test or the Mann-Whitney test, depending on whether the distribution was normal or not. Correlations were estimated with the Pearson method. Finally, multiple regression was applied. HOMA-IR was the dependent variable using a stepwise backward selection of the explicative variables. HOMA-IR was logarithmic transformed before its use. Each predictor contribution was assessed by studying its significance (α-level, 0.05). Statistical analyzes were performed with SPSS 22 (IBM, Armonk, New York).

## Results

### OSA and non-OSA patients’ characteristics

In Table 1 the general characteristics of the total population and of the OSA and non OSA patients are listed.

**Table 1:** Patients’ characteristics.

	Total population	Non OSA group (n = 25)	OSA group (n = 47)	P-value
Age (yrs)	74.5±7.8	73.1 ± 7.1	75.2 ± 8.1	0.253^¶^
Gender M/F	34/38	12/13	22/25	0.923^†^
BMI (kg/m^2^)	25.9±4.6	24.3 ± 4.5	26.8 ± 4.4	**0.028**^¶^
Neck circumference (cm)	38.6±4.1	36.6 ± 3.1	39.7 ± 4.3	**0.001**^¶^
Waist circumference (cm)	95.8±13.6	90.3 ± 14.5	98.9 ± 12.2	**0.013**^‡^
Hypertension n = (%)	38 (52.8%)	15 (60%)	23 (49%)	0.420^†^
Systolic BP (mmHg)	139.9±18.3	140.2 ± 18.8	139.8 ± 18.3	0.924^¶^
Diastolic BP (mmHg)	78.8±10.2	79.2 ± 9.8	78.6 ± 10.4	0.990^‡^
Dyslipidemia n = (%)	24 (33.3%)	10 (40%)	14 (30%)	0.416^†^
Stroke n = (%)	7 (9.7%)	3 (12%)	4 (8.5%)	0.656^†^
Heart disease n = (%)	12 (16.7%)	4 (16%)	8 (17%)	0.881^†^
Atrial Fibrillation n = (%)	5 (6.9%)	1 (4%)	4 (8.5%)	0.460^†^
Hypothyroidism	14 (19.4%)	4 (16%)	10 (21%)	0.562^†^
MMSE	25±4.7	26.1 ± 4.1	24.4 ± 4.9	0.177^¶^
GDS	9.5±6.1	10.6 ± 5.8	8.9 ± 6.3	0.297^‡^
**Plasma glucose/insulin homeostasis**				
Fasting Glucose (mmol/L)	5.32±0.56	5.3 ± 0.6	5.3 ± 0.5	0.403^‡^
Fasting Insulin (mUI/L)	8.9±6.3	6.6 ± 3.7	10.1 ± 7.0	**0.010**^‡^
HOMA-IR	2.2±1.7	1.5 ± 0.9	2.5 ± 1.9	**0.014**^‡^
HbA1C (%)	5.8±0.4	5.8±0.3	5.8±0.4	0.249^‡^
HbA1C (mmol/mol)	39.6±0.5	39.9±0.7	39.4±0.7	0.298^‡^
IGF-1 (μg/L)	144,35±49.8	145.5 ± 56.7	143.7 ± 45.8	0.896^¶^
IGF-1 score	0.42±1.40	0.29 ± 1.60	0,49 ± 1.29	0.598^¶^
**Plasma lipid profile**				
Cholesterol total (mmol/L)	5.3±1.0	5.2 ± 1.0	5.4 ± 1.0	0.504^¶^
HDL-cholesterol (mmol/L)	1.44±0.3	1.6 ± 0.3	1.4 ± 0.3	**0.033**^¶^
LDL-cholesterol (mmol/L)	3.2±0.8	3.1 ± 0.9	3.2 ± 0.8	0.893^¶^
Triglycerides (mmol/L)	1.3±0.5	0.9 ± 0.3	1.5 ± 0.4	**0.000**^¶^
**Sleep subjective characteristics**				
Snoring n = (%)	43 (59.7%)	12 (48%)	31 (66%)	0.106^†^
Subjective sleepiness n = (%)	25 (34.7%)	6 (24%)	19 (40%)	0.102^†^
ESS	7.4±5.3	5.7 ±3.4	8.3 ± 5.9	**0.026**^¶^
Observed apnea n = (%)	17 (23.6%)	3 (12%)	14 (30%)	**0.043**^†^
Nocturnal polyuria n = (%)	47 (65.3%)	12 (48%)	35 (74%)	**0.031**^†^
Fatigue n = (%)	43 (59.7%)	16 (64%)	27 (57%)	0.665^†^
Morning headaches n = (%)	17 (23.6%)	6 (24%)	11 (23%)	0.958^†^
Diary reported sleep time (hrs)	5.9 ±2.1	5.2±1.4	6.2±2.3	0.164^‡^
**Objective sleep characteristics (polysomnography)**				
TST (min)	337.5±79.1	332.4 ± 92.3	340.2 ± 73.4	0.713^¶^
Sleep efficiency (%)	69.5±14.5	68.9 ± 17.5	69.8 ± 12.9	0.824^¶^
SOL (min)	23.8±28.4	25.8 ± 36.2	22.9 ± 24.0	0.712^‡^
WASO (min)	119.1±73.3	105.5 ± 73.1	126.4 ± 73.1	0.254^‡^
Sleep stage I (%TST)	27±15.2	18.7 ± 12.6	31.3 ± 14.7	**0.000**^‡^
Sleep stage II (%TST)	40.6±12.1	43.5 ± 12.4	38.9 ± 11.7	**0.047**^‡^
Sleep stage III (%TST)	16.5±8.9	20.3 ± 8.8	14.5 ± 8.3	**0.009**^‡^
REM sleep stage (% TST)	16.4±10.6	17.3 ± 7.4	15.9 ± 11.9	0.147^‡^
Arousals/hour of sleep	28.4±17.1	18.5 ± 10.6	33.6 ± 17.6	**0.000**^‡^
AHI (events/hr)	25.8±18.2	8.3 ± 4.3	35.1 ± 15.7	**0.000**^‡^
Mean SpO_2_ (%)	93.2 ± 2.3	93.9 ± 2.5	92.8 ± 2.2	0.078^¶^
Time spent <90% of SpO_2_ (%)	9.5±17.8	7.2 ± 19.9	10.5 ± 16.8	**0.002**^‡^
MSLT (min)	11.8±4.8	12.0 ± 4.1	11.6 ± 5.2	0.699^¶^

Notes: Mean ± SD are given. Abbreviations: yrs, years; M, male; F, female; BMI, body mass index; BP, Blood Pressure; MMSE, Mini-mental state examination; GDS, Geriatric Depression Scale; HOMA-IR, HOMA Insulin Resistance Index; ESS, Epworth Sleepiness Scale; Hrs, hours; TST, total sleep time; SOL, sleep onset latency; WASO, waking after sleep onset; REM, rapid eyes movement; AHI, apnea-hypopnea index; MSLT, multiple sleep latency test.

^¶^ Student’s t-test

^†^ Pearson’s χ^2^ test

^‡^ Mann-Whitney test; P-values indicating significance are shown in bold type

#### Clinical findings in OSA and non OSA patients

Mean age was 74.5 ± 7.8 years (60 to 93 years). Women were 52.8% of the total sample. The mean BMI was 25.9 ± 4.6 kg/m^2^. Anthropometric measures were significantly different: OSA patients presented higher BMI, neck and waist. After excluding obese patients (BMI ≥ 30kg/m^2^; n = 15), the mean BMI was 24.1±3.05 kg/m^2^ and there were still significant differences in BMI (p = 0.016) and neck circumference (p = 0.006) between OSA and non OSA patients.

Polypathology and polypharmacy, highly prevalent in the older patients, were also evaluated. No differences regarding, blood pressure, MMSE, GDS were found. Also co-morbidities and the use of medications such as benzodiazepine receptor agonists, benzodiazepines, cardiovascular treatment, statins, and fibrates were not statistically different in the two groups. Medications with potential effects on insulin sensitivity and carbohydrate metabolism (statin, beta–blocker, sartan, calcium antagonist, diuretics, hydrochlorothiazide, angiotensin-converting-enzyme inhibitor) showed no significant difference between OSA and non OSA groups. No one has taken steroid therapy (results not shown).

#### Biological findings in OSA and non OSA groups: IR is more prevalent in OSA patients

While glycaemic control (fasting blood glucose and glycated hemoglobin) was the same in both groups, IR (fasting insulin and HOMA-IR) and triglycerides were higher in OSA patients, while HDL-cholesterol was lower. Total and LDL-cholesterol, hepatic and thyroid function, creatinine and nutritional status (data not shown) and IGF-1 level and IGF-1 score were not statistically different (Table 1).

Comparing moderate and severe OSA, there was a significant difference in HOMA-IR between moderate OSA and severe OSA patients (1.8±1.0 *vs* 3.1±2.4; p = 0.032): severe OSA patients showed more insulin resistance (data not shown).

Moreover, the HOMA-IR did not differ between men and women in our study (data not shown).

#### Sleep parameters in OSA and non OSA patients

As some sleep parameters, such duration, quality and fragmentation of sleep, snoring and daytime sleepiness could be involved in IR pathophysiology, we have studied these other potential confounding factors in our population. Subjective sleep complaints are known to be potential symptoms of OSA, and our OSA patients reported more nocturnal polyuria and observed apneas. There were no differences in reported snoring, subjective daytime excessive sleepiness, fatigue and morning headaches between the two groups. ESS was significantly higher in OSA patients, but without clinical significance because the absolute score was <10, therefore too low to define a sleepiness state (Table 1).

Regarding objective sleep recording, OSA patients presented a mean AHI of 35.1 ± 15.7/hr, indicating moderate to severe OSA. There were 23 patients with moderate OSA and 24 with severe OSA. They presented more arousals per hour of sleep and a longer time spent under 90% of SpO2. Mean nocturnal SpO2 was not significantly different between non OSA and OSA groups, but was different when compared between non OSA and severe OSA group (p = 0.012). Total sleep time (TST), sleep efficiency, sleep onset latency (SOL), waking after sleep onset (WASO) and mean sleep latency (MSL) in the MSLT were not different between non OSA and OSA groups, and also between non OSA and severe OSA groups. Compared to controls, OSA patients had longer sleep stage I, and shorter sleep stages II and III. However no difference in REM sleep duration was detected (Table 1).

### Sleep parameters in patients with and without insulin resistance

As the association of some sleep parameters with IR was reported in other studies, we have analyzed the link between sleep parameters and IR in our patients. IR patients had higher AHI, lower mean SpO2, and longer time spent under 90% of SpO2, parameters which are related to OSA severity. Sleep stage 1 and arousals/hour of sleep were also higher in the IR group (Table 2).

**Table 2:** Sleep parameters in patients with and without insulin resistance.

	Total population	Non IR group HOMA-IR<2.5 (n = 48)	IR group HOMA-IR≥2.5 (n = 24)	P-value
**Plasma glucose/insulin homeostasis**				
HOMA-IR	2.2±1.7	1.3±0.5	3.9±2.0	0.000^‡^
**Sleep characteristics**				
ESS	7.4±5.3	7.4±5	7.3 ± 5.9	0. 916^¶^
TST (min)	337.5±79.1	335.4 ± 75.4	341.7 ± 89.8	0.758^¶^
Sleep efficiency (%)	69.5±14.5	69.1 ± 13.5	70.4 ± 16.7	0.466^‡^
SOL (min)	23.8±28.4	27.3 ± 32.3	17.1 ±17.6	0.220^‡^
WASO (min)	119.1±73.3	118.9 ± 65.7	119.5 ± 88.1	0.526^‡^
Sleep stage I (%TST)	27±15.2	23.9 ± 14.7	33.1 ± 14.6	**0.009**^‡^
Sleep stage II (%TST)	40.6±12.1	41.5 ±12.6	38.7 ± 11.1	0.352^‡^
Sleep stage III (%TST)	16.5±8.9	17.6 ± 9.1	14.4 ± 8.2	0.151^‡^
REM sleep stage (% TST)	16.4±10.6	17.8± 11.8	13.6± 7.0	0.100^‡^
Arousals/hour of sleep	28.4±17.1	25.3 ± 16.2	34.7 ± 17.4	**0.029**^‡^
AHI (events/hr)	25.8±18.2	20.5 ±13.7	36.5 ± 21.5	**0.001**^‡^
Mean SpO2 (%)	93.2 ± 2.3	93.9±2.2	91.9±2.2	**0.001**^¶^
Time spent under 90% of SpO2 (%)	9.5±17.8	7.1 ± 16.5	14.3 ± 19.7	**0.001**^‡^
Mean sleep latency in MSLT (min)	11.8±4.8	12.0 ± 4.8	11.1 ± 4.9	0.484^¶^

Notes: Mean ± SD are given. Abbreviations: HOMA-IR, HOMA Insulin Resistance Index; ESS, Epworth Sleepiness Scale; MSLT, multiple sleep latency test; TST, total sleep time; SOL, sleep onset latency; WASO, waking after sleep onset; REM, rapid eyes movement; AHI, apnea-hypopnea index. P-values indicating significance are shown in bold type.

¶ Student’s t-test

‡ Mann-Whitney test

### Sleep parameters are not associated with IGF-1

As some sleep parameters were associated with IR, we studied the correlation of sleep parameters on IGF-1 level and IGF-1 score. IGF-1 levels correlated with subjective sleepiness Epworth scale (p = 0.010) but not with objective sleepiness (mean sleep latency on MSLT). Age-adjusted IGF-1 score was only correlated with the ESS (p = 0.018). Others parameters such as AHI, TST, and sleep stages were not correlated with either IGF-1 level or IGF-1 score.

### OSA is independently associated with IR

**Fig 1:**
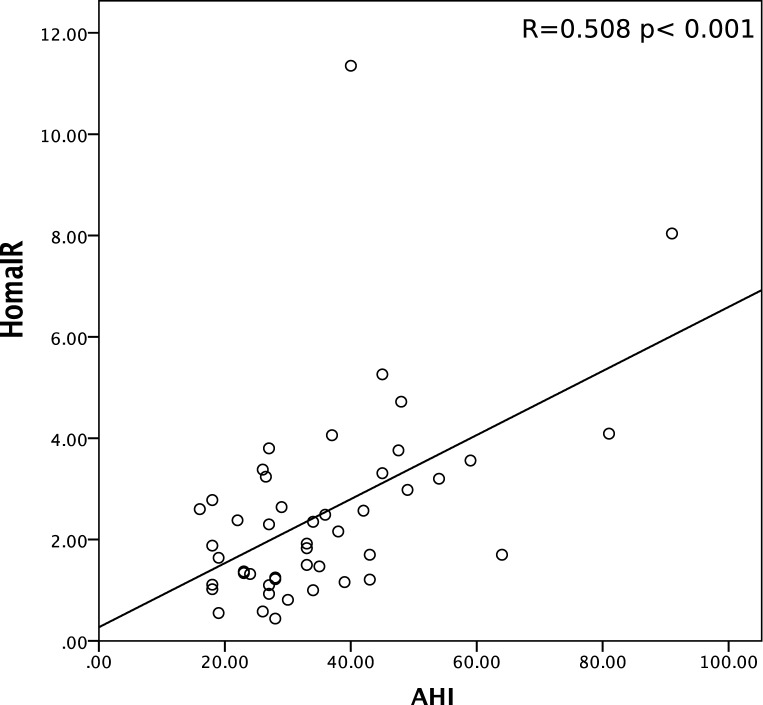
Scatter plot of apnea–hypopnea index (AHI) and HOMA-IR in OSA group.

HOMA-IR is significantly more elevated in the OSA group than in the non OSA group (Table 1, p = 0.014). To examine the association between OSA and IR, we analyzed the relationship between AHI and HOMA-IR. No significant correlation was found in the non OSA group (R = 0.221, p = 0.288). By contrast, we observed a significant positive correlation between AHI and HOMA-IR in the OSA group (R = 0.508, p < 0.001) (Fig 1).

Since our OSA patients differed from controls by anthropometric measures and some sleep parameters, multilinear regression analysis was used to look for the variable associated independently with IR in the total population (Table 3). In models using HOMA-IR as the dependent variable, the analysis showed that AHI was independently associated with HOMA-IR in the total population (B = 0.009 (0.004), β = -0.062, p = 0.027). Anthropometric measures, TST, nocturnal arousals (arousals/h of sleep), objective (MSLT) and subjective sleepiness (ESS), nocturnal hypoxemia (time spent under 90% of SpO2), and sleep stages I and III were not associated with IR. In the OSA group, the independent association between AHI and HOMA-IR remained (B = 0.015 (0.005), β = 0.683, p = 0.011).

**Table 3:** Summary of multiple linear regression for screening variables predicting on HOMA-IR in total population.

	B	:± SE	β	p Value
BMI	0.017	0.017	0.176	0.327
Neck	0.020	0.015	0.163	0.183
Hips	0.004	0.009	0.152	0.648
Waist	0.001	0.008	0.527	0.912
AHI	0.009	0.004	-0.062	0.027*
TST (mn)	0.000	0.001	-0.193	0.797
Arousals/h	-0.002	0.005	-0.089	0.692
MSLT	-0.004	0.010	-0.008	0.674
ESS	-0.001	0.010	-0.041	0.889
Time spent under 90% of SpO2	0.000	0.003	0.012	0.919
Stage I	0.000	0.005	0.176	0.949
Stage III	0.000	0.002	-0.008	0.959

Note: Transformation of Homa IR was performed using LN (HOMA IR). Overall model was significant (F_11, 46_ = 4.27, **p Value <0.0001**, R2 = 77.6).

*P< 0.05; P-values indicating significance are shown in bold type.

### IGF-1 score is not related to insulin resistance observed in OSA

The putative role of IGF-1 in the insulin resistance observed in OSA was also examined.

IGF-1 and IGF-1 score were not different between OSA and non OSA patients (Table 1). AHI was not also correlated to IGF-1 level or IGF-1 score (p = 0.998 and p = 0.593 respectively). Moreover, by analyzing IGF1 and IGF1 score in patients with and without IR, by using a HOMA-IR cut-off corresponding to the median (1.7) and another cut-off at 2.5, there was no difference in IGF-1 or IGF-1 score between patients with and without IR (data not shown). Finally in the Spearman’s correlation analysis, there was no correlation between HOMA-IR and IGF-1 score in OSA and non OSA groups (p = 0.066 and p = 0.380 respectively; data not shown).

## Discussion

We describe here the independent association between OSA and IR in a non-diabetic older population with sleep complaints, using full-night PSG to characterize breathing abnormalities during sleep, and considering confounding factors such as sleep parameters. In addition, IGF-1 level and age-adjusted IGF-1 score were not associated with OSA in our study and the association between OSA and IR in our cohort was independent of IGF-1.

### Anthropometric measures and OSA

Although in a middle-aged population OSA is strongly associated with obesity, our older patients with OSA were over-weight but not obese (mean BMI = 26.8 ± 4.4 kg/m^2^). This is in line with another study where the association between BMI and AHI was weaker in older than in younger patients [21], perhaps partially due to changes in parapharyngeal structure anatomy [22]. In clinical practice, our data show that screening for OSA should not be restricted to the obese among these older patients.

### IR and OSA

OSA patients were more insulin resistant than non OSA patients. Moreover, severe OSA patients had a higher HOMA-IR than moderate OSA patients. The multilinear regression model demonstrated that OSA is independently associated with IR: AHI was independently associated with IR, after adjustment for several confounding factors including anthropometric and other objective sleep measures. This association has been already observed in younger patients with the use of PSG [4]. In a mixed population (middle-aged and older community-based patients), this association has been suggested, but OSA was not objectively evaluated by PSG and only intermittent hypoxia was evaluated by a nocturnal oximetry [7]. Apneas and hypopneas lead to nocturnal hypoxia and sleep fragmentation. In the Sleep Heart Health Study, sleep fragmentation, as indicated by recurrent cortical arousals, was associated with IR [5]. A recent animal study has shown that sleep fragmentation can induce IR in mice, through activation of oxidative stress and inflammation [23]. In our study, even though the frequency of arousals was high in OSA patients, it was not independently associated with IR, unlike AHI. This suggests that intermittent apneas and hypopneas are sufficient to lead to IR in older non-diabetic patients, regardless of the severity of arousals. Other factors, such as enhanced sympathetic activity, are known to be implicated in IR induced by OSA [24]; however, since we did not evaluate these parameters, we cannot speculate on the mechanisms inducing IR in our older OSA non-diabetic patients.

### IGF-1, sleep, and OSA

IGF-1 plays a role in the pathophysiology of IR [14, 23]. Data about the link between OSA and IGF-1 level are variable and seem to depend on the age of the population studied. In older patients, OSA did not appear to adversely influence the GH/IGF axis, contrary to what is reported in younger patients [17]. Indeed, in younger but obese patients, the positive association between low IGF-1 levels and OSA is well-established [15]. Furthermore, in a middle-aged population, treatment of OSA seems to increase the IGF-1 level [25]. In our study, neither IGF-1 level nor age-adjusted IGF-1 score was different in older non-diabetic patients, with or without OSA. In Japanese primary snorers aged ≥60 years, there were also no differences in HOMA-IR or in IGF-1 levels between OSA and non-OSA subjects [26]. Altogether these results do not support the hypothesis that the association of IR and OSA involves the IGF-1 system, at least in aged patients.

Concerning sleep parameters, our correlation study showed that IGF-1 and age-adjusted IGF-1 score were correlated with Epworth (p = 0.010 and p = 0.018 respectively), but not with AHI, MSLT, or others sleep variables. A previous study in older patients showed no association between objective sleep parameters and IGF-1, but daytime sleepiness was not analyzed [17]. Our study is the first to analyze objective daytime sleepiness by MSLT and measured IGF-1. We additionally took into account the age effect on IGF-1 level, by analyzing age-adjusted IGF-1 score.

### IR and sleepiness

It has been reported that both subjective and objective daytime sleepiness were associated with IR in middle-aged sleep apnea patients, independently of obesity [27]. In our older patients, neither ESS nor MSLT were associated with IR. Although OSA patients presented higher ESS score than non-OSA patients, the absolute value of ESS was not pathological (ESS<10), and not defining subjective sleepiness. However, ESS is not suitable for evaluating sleepiness in older people [28], and we therefore analyzed the objective sleepiness with MSLT.

### IR and sleep duration/stage

Recent studies have underlined the association of sleep complaints and sleep duration with IR in non-diabetic middle-aged patients [12,13]. In our study, all the patients were referred to our hospital for sleep complaints, but only AHI was independently associated with IR, not the objective sleep duration (TST). Some studies have reported that loss of sleep quality contributes to glucose metabolism impairment. Indeed, suppression of slow-wave sleep during 3 nights induced impairments in glucose metabolism in humans [29]. Our OSA patients presented shortened sleep stages II and III (slow-wave sleep), because of nocturnal arousals increasing sleep stage I. However in multiple linear regression analysis, the times of sleep stages I and III were not associated with HOMA-IR.

### Perspectives

Patients with IR are at increased risk of developing both cardiovascular diseases and type 2 diabetes mellitus, two major health problems [30]. Moreover, older patients with IR may be at higher risk of developing adverse geriatric syndromes such as sarcopenia and frailty [31]. Recent meta-analyses have assessed the effect of CPAP therapy on IR in non-diabetic patients with OSA. These analyses included both uncontrolled studies and randomized-controlled trials, but overall they suggested some reduction in HOMA-IR with medium-term use of CPAP, at least among compliant patients [9]. Further studies are needed to determine whether therapeutic approaches targeting OSA may represent a novel strategy to preserve or recover insulin sensitivity in older subjects.

The strengths of this study performed in an older population include the use of full night PSG, the gold-standard method for sleep study and diagnosis of OSA; objective measures of IR (evaluated without antidiabetic treatments interfering with HOMA-IR); use of age-adjusted IGF-1 score; objective evaluation of daytime sleepiness (MSLT); and the inclusion of numerous confounding covariates (e.g. anthropometric measures, several objective sleep parameters). However, the use of no random sampling method, the non population-based nature of this study, and the relatively small sample size without performing a power analysis prior to data collection limit the availability to generalize results. Statistical power in some significant quantitative variables was nevertheless performed (power of 82% or 100%; alpha = 0.05, two-tail). Larger confirmatory studies are needed before suggesting treatment of OSA as a novel strategy to preserve or recover insulin sensitivity in the older subjects.

## Conclusions

OSA is independently associated with IR. We have shown for the first time that this association, in older non-diabetic patients with sleep complaints, is independent of anthropometric measurements and other sleep parameters. In our population, it seems that IGF-1 axis did not play a role in IR association with OSA. IR predicts mortality in older patients and is associated with cardiovascular and metabolic diseases, and with geriatric syndromes [31]. As OSA is treatable and its treatment improves IR, therapeutic strategies reducing IR may be useful to prevent such bad outcomes. Its role is worthy of further exploration in older people.

## Abbreviations

ALT: Alanine aminotransferase
AHI: Apnea-Hypopneas Index
AST: Aspartate aminotransferase
C-PAP: Continuous Positive Airway Pressure
BMI: Body Mass Index
GDS: Geriatric Depression Scale
EEG: Electroencephalography
ESS: Epworth Sleepiness Scale
fT4: Free thyroxine
fT3: free triiodothyronine
GGT: Gamma-glutamyl transferase
GH/IGF-1: Growth Hormone/Insulin like growth factor 1
HDL: High Density Lipoprotein
HOMA-IR: Homeostatic Model Assessment for IR
IR: Insulin Resistance
MMSE: Mini-Mental State Examination
MSL: Mean Sleep Latency
MSLT: Multiple Sleep Latency Tests
OSA: Obstructive Sleep Apnea
ODI: Oxygen desaturation index
PSG: Polysomnography
REM: Rapid Eyes Movement
SOL: Sleep Onset Latency
TSH: Thyroid Stimulating Hormone
TST: Total Sleep Time
WASO: Waking After Sleep Onset
